# An *In Vitro* Model of Skeletal Muscle Volume Regulation

**DOI:** 10.1371/journal.pone.0127889

**Published:** 2015-06-01

**Authors:** Anna Wibberley, Caroline A. Staunton, Claire H. Feetham, Alexey A. Vereninov, Richard Barrett-Jolley

**Affiliations:** 1 Department of Musculoskeletal Biology, Institute of Ageing and Chronic Disease, University of Liverpool, Liverpool, United Kingdom; 2 Laboratory of Cell Physiology, Institute of Cytology, Russian Academy of Sciences, St-Petersburg, Russia; Albany Medical College, UNITED STATES

## Abstract

**Introduction:**

Hypertonic media causes cells to shrink due to water loss through aquaporin channels. After acute shrinkage, cells either regulate their volume or, alternatively, undergo a number of metabolic changes which ultimately lead to cell death. In many cell types, hypertonic shrinkage is followed by apoptosis. Due to the complex 3D morphology of skeletal muscle and the difficulty in obtaining isolated human tissue, we have begun skeletal muscle volume regulation studies using the human skeletal muscle cell line TE671RD. In this study we investigated whether hypertonic challenge of the human skeletal muscle cell line TE671RD triggered cell death or evoked a cell volume recovery response.

**Methods:**

The cellular volume of TE671RD cells was calculated from the 2D surface area. Cell death was assessed by both the trypan blue live/dead assay and the TUNEL assay.

**Results:**

Medium osmolality was increased by addition of up to 200mM sucrose. Addition of 200mM sucrose resulted in mean cell shrinkage of 44±1% after 30mins. At later time points (2 and 4 hrs) two separate cell subpopulations with differing mean cell volume became apparent. The first subpopulation (15±2% of the total cell number) continued to shrink whereas the second subpopulation had an increased cell volume. Cell death was observed in a small proportion of cells (approximately 6-8%).

**Conclusion:**

We have established that a substantial proportion of TE671RD cells respond to hypertonic challenge with RVI, but that these cells are resistant to hypertonicity triggered cell death.

## Introduction

Regulatory volume increase (RVI) and apoptotic volume decrease (AVD) are two opposing cellular volume-regulatory mechanisms [1–3]. The first of these, RVI, is frequently involved with adaptation to hypertonic media and cell survival, whilst in some cells, but not others AVD leads to cell death [4, 5]. Frequently after cells reach a certain critical threshold of shrinkage, cells then undergo RVI or AVD. Hypertonic challenge can lead to apoptosis in a number of cell types [6–10]. In this study we investigate whether hypertonic challenge induces cell death in a human derived skeletal muscle cell line TE671RD.

Overall control of systemic osmolality is affected by a number of periventricular osmosensing structures within the brain [11] and involves osmotic response of individual neurones by mechanisms analogous to that of cell volume regulation itself [12]. Older people have an approximately 3% (302.2 compared with 291.2 mOsm/Kg H_2_O) increased plasma osmolality compared to healthy younger people [13]. This could be due to changes in kidney function, as a result of hypertension, or due to environmental factors such as diet. However, a loss in osmotic response is also observed in the elderly, suggesting that an issue with osmotic control could result in increased plasma osmolality [14, 15]. Cells are generally able to withstand small (2–3%) changes in tissue osmolality, but beyond this the activation of volume defence mechanisms becomes necessary [16]. Such chronic change in plasma osmotic potential of older people could therefore have a negative impact on skeletal muscle physiology, affecting such parameters as cellular volume. Indeed, a number of genes critical to both cell volume control and apoptosis, including the AQP2 and AQP3 aquaporin channels, are differentially expressed in ageing skeletal muscle [17]. The importance of apoptosis to ageing skeletal muscle physiology is controversial. It has been argued that apoptosis is not necessarily pathological and is important for the process of remodelling [18], but it is increased subtly during ageing [19]. It has therefore been hypothesised that apoptosis may contribute to sarcopenia in older people [19–22], potentially resulting from mitochondrial dysregulation [23]. Different skeletal muscle fibre types have been shown to have differing propensity to undergo apoptosis in response to TNFα [24], but apoptosis in response to hypertonic challenge has not previously been investigated. TE671RD cells are potentially an ideal model for cell volume regulation experiments because whilst they derive from human skeletal muscle they readily round-up and facilitate volume measurement. There is no evidence for the presence of t-tubules in TE671RD cells, structures which would naturally confound simple geometric estimation of cellular volume in native skeletal muscle fibres. The literature shows that TE671RD cells express the skeletal muscle specific form of the nicotinic acetylcholine receptor [25] and TTX-resistant voltage-gated sodium channels characteristic of native skeletal muscle [26]. Another ion channel, however, the K_ATP_ channel is not pharmacologically identical in TE671RD cells [27] to that found in our own rat skeletal muscle studies [28]. It should be noted, however, that most skeletal muscle ion channel studies use rodent or amphibian tissue rather than human and so species is a confounding variable. Aside from muscle ion channel expression, a recent study showed the expression of striated muscle developmental micro mRNA mir206 in TE671RD cells [29–31], but there have been few studies of other muscle like properties such as expression of contractile apparatus. There are also very limited data available on their volume regulation.

In this study we analysed whether severe hypertonic challenge triggers RVI and/or cell death in TE671RD. We find that whilst there is a consistent reduction in cell volume in response to hypertonic challenge and strong evidence of RVI very little cell death occurs and it does not seem likely that this will contribute to any loss of motor units in the elderly.

## Methods

### Culture

TE671RD cells constitute a human rhabdomyosarcoma cell line showing features of skeletal muscle differentiation [32, 33]. These were a kind gift from the group of Prof Vincent (Oxford University, [34]). TE671RD cells were maintained in 300mOsm/Kg H_2_O DMEM (GIBCO 31885, Life Technologies Ltd, Paisley, UK) medium with 10% fetal calf serum, 1% amphotericin B and 2% penicillin and streptomycin at 37°C and 5% CO_2_. All reagents were obtained from Sigma-Aldrich (UK) unless otherwise stated.

### Measurement of cell size

Cells were detached from the flask surface by 0.25% Trypsin-EDTA solution. A pellet was formed by centrifugation of the cell solution (300 x g, 5 min) (MultifugeX1, Thermo Fisher Scientific) and supernatant removed. Cells were then re-suspended in culture media with FCS and incubated for 1 hour at 37°C and 5% CO_2_ before the experiment. We measured cell shrinkage upon exposure to hypertonic media with a wide range of sucrose concentrations (0–200 mM). Osmolality was measured using a freezing point micro-osmometer (3MO, Advanced Instruments, Norwood, USA). We compared cell volume in isotonic control and hypertonic samples using light microscopy with an Olympus IX51 equipped with a JVC TK-C921EG digital camera. Cell volume was calculated as described previously [35] from the 2D areas with ImageJ software [36].

### Cell viability

Cell viability was assessed with the trypan blue exclusion assay. DNA fragmentation characteristic of apoptotic programmed cell death was detected with the terminal deoxynucleotidyl transferase (TdT)-mediated dUTP nick end labelling (TUNEL) assay [37] using the Trevigen apoptotic cell system (TACS) TACS 2 TdT-Fluor Apoptosis Detection Kit (Trevigen, Gaithersburg, MD, USA). Control and sucrose treated cells were fixed in 10% neutral buffer formalin (NBF), placed onto slides and then processed according to the manufacturer’s instructions. After staining, slides were mounted with DAPI enriched VECTASHIELD Mounting Medium. Statistical analysis was carried out using the non-parametric Mann-Whitney test (p ≤ 0.05) in MiniTab (Minitab Ltd., Coventry, UK).

## Results

**Fig 1:**
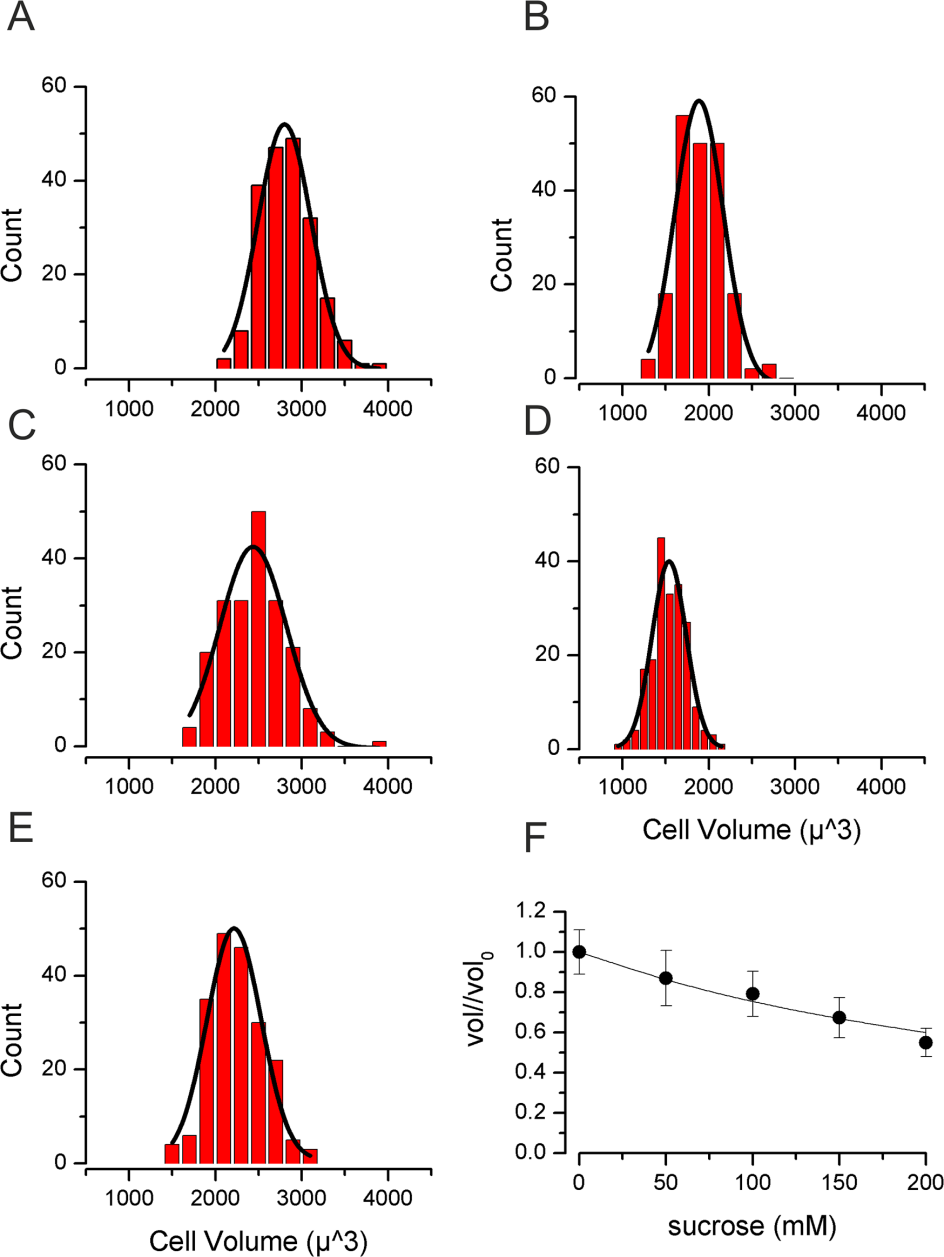
Volume of TE671RD following 30 min of increased osmolality. **(A)** Cells measured in 300mOsm/Kg H_2_O (no sucrose), **(B)** with 350 mOsm/Kg H_2_O (50 mM sucrose), **(C)** with 400 mOsm/Kg H_2_O (100mM sucrose) **(D)** with 450 mOsm/Kg H_2_O (150mM sucrose), **(E)** with 500 mOsm/Kg H_2_O (200mM sucrose). For (A) to (E) smooth lines are Gaussian fits. **(F)** Normalised volume against sucrose concentration. The smooth line is drawn to that of ideal osmotic behaviour: *Vol*/*Vol*_0_ = *V*_0_ * *osm*_0_/*osm*_*sucrose*_. Where *Vol*_*o*_ and *Vol* are the initial volume and the volume attained by the cells in the sucrose containing solution (measured at 30mins). *osm*_*0*_ is the initial osmolality of the solution (I.e., DMEM alone, 300mOsm/Kg H_2_O) and *osm*_*sucrose*_ is the osmolality once sucrose had been added (i.e., 300mOsm/Kg H_2_O + [sucrose]).

**Fig 2:**
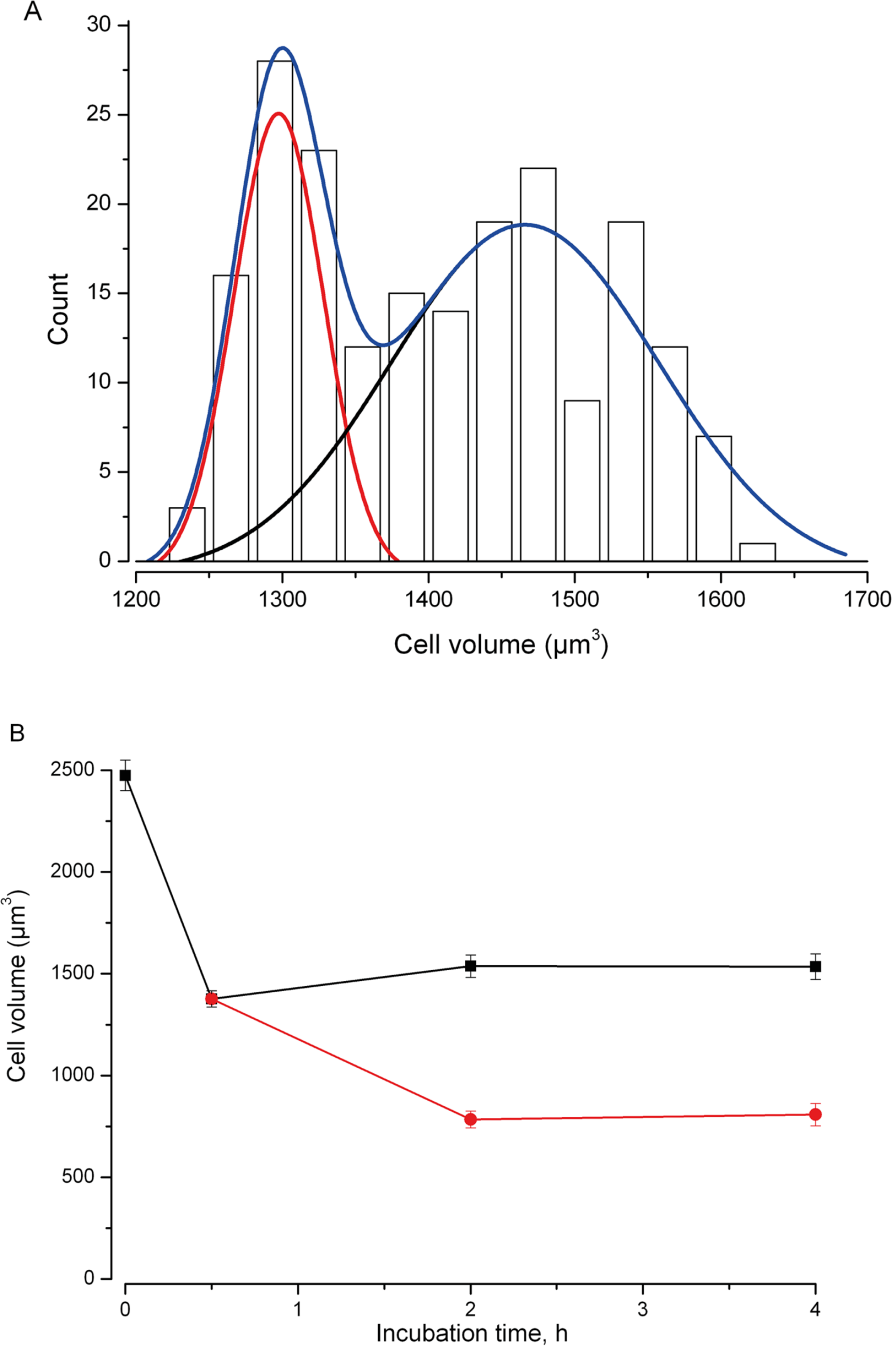
Differential volume defence mechanisms within TE671RD populations. Following prolonged exposure to 200mM sucrose (osmolality 500mOsm/Kg H_2_O), two populations of cells were distinguishable. **(A)** Illustrates one experiment when cells have been exposed to 200mM sucrose (osmolality 500mOsm/Kg H_2_O) for 2hrs. Two populations are clearly apparent. The blue line illustrates a 2 component Gaussian fit to the data with smaller and larger components indicated by red and black lines respectively. **(B)** Data from between 6 and 13 experiments similar to that illustrated in (A). The first population continued to shrink (red circles); the second population of cells (black squares) maintained or increased volume. At 2 hrs 15±2% (n = 9) of cells are included in the smaller population, at 4hrs 17±2% (n = 7) are included in the smaller population.

Under iso-osmotic control conditions (300 mOsm/Kg H_2_O) mean TE671RD cell volume was 2840±22μm^3^ (*n* = 200, Fig 1). Following 30 minutes incubation with sucrose (0 to 200mM), volume decreased to close to that predicted from “ideal osmotic behaviour” [38] (Fig 1). We then went on to measure cell volume at a series of time points (0.5, 2 and 4hr) following exposure to 200mM sucrose. We measured cell volume in between 100 and 200 cells in each of 13 separate experiments. Initially volume fell to 44±1% (n = 13, t = 0.5hr). By 2hrs cells had clustered into two distinct populations (assessed by cellular volume). Those cells identified in the first population reduced in size, whereas cells of the second population significantly increased in cellular volume (Fig 2). At 2hr 18±1% (n = 8) of cells had increased their volume (I.e., exhibited RVI) and this rose to 21±1% (n = 6) of cells at 4hr.

**Fig 3:**
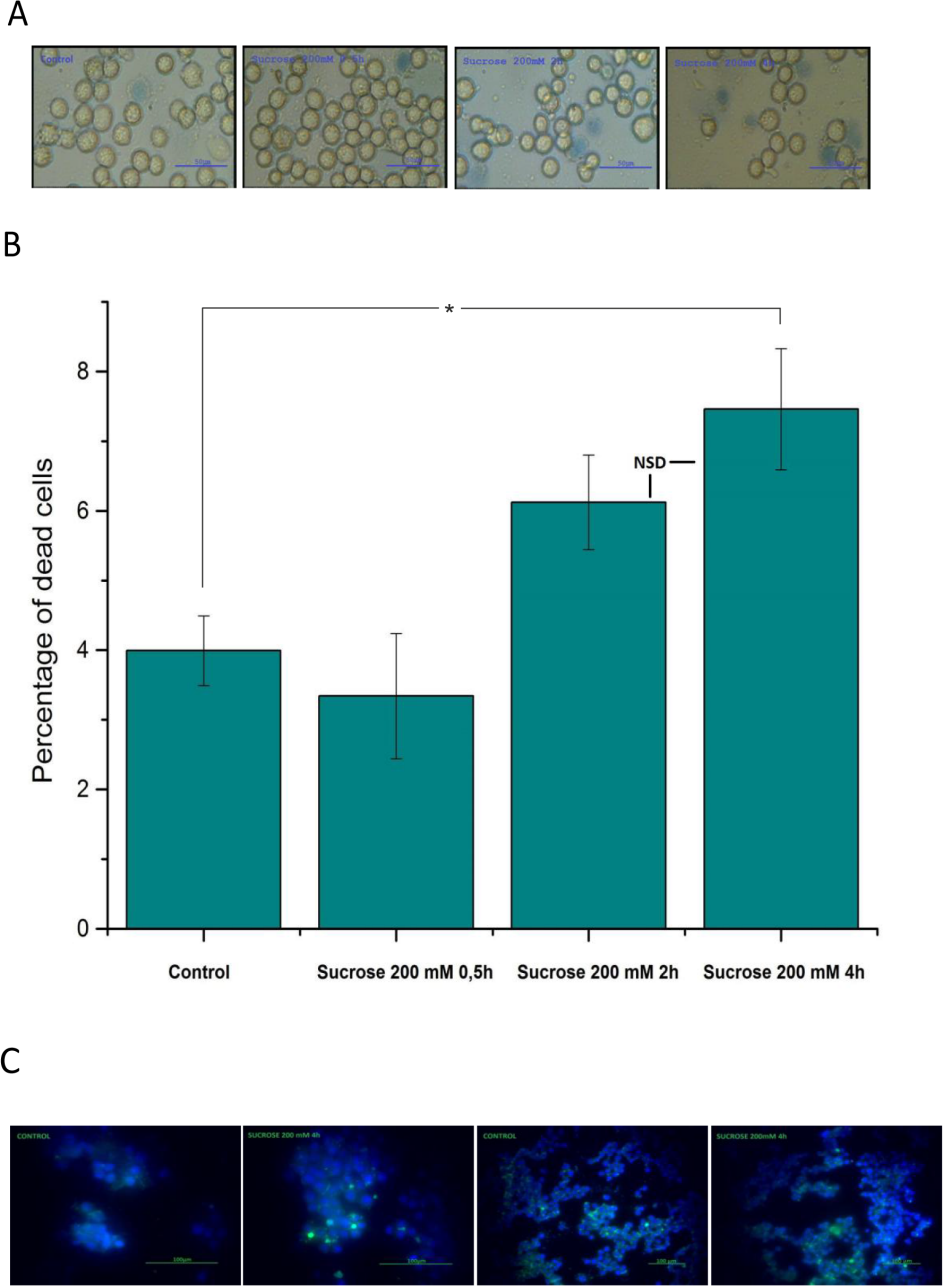
Cell viability assays. **(A)** Example of the live/dead trypan blue exclusion assay. Cells are shown in control and then during 0.5h-4h incubation in hypertonic media with 200 mM/Kg H_2_O sucrose (from left to right). **(B)** Quantification of the live/dead trypan blue exclusion assay with time. Cell death with 200mM sucrose at 2hrs and 4hrs was statistically increased from control (p<0.05, Mann-Whitney), but there was no statistical difference between 2hrs and 4hrs. **(C)** Fluorescent images of TE671RD cells stained by DAPI (blue) and TACS 2 TdT-fluor “Apoptosis Detection Kit” (green) obtained using Nikon Eclipse microscope. Fluorescent microscopy showed the presence of DNA fragmentation after 4 h of 200 mM sucrose incubation. 6±1% (n = 5) of sucrose-treated cells demonstrated a bright green nucleus with condensed chromatin. The TUNEL apoptosis assay was conducted at 4hrs.

Cell shrinkage can be a trigger for apoptosis in many cell types and so we investigated hypertonicity evoked changes in cell viability and DNA fragmentation (an indicator of apoptotic programmed cell death) using the trypan blue exclusion assay and TUNEL assay respectively. We found an increase of dead cell count after prolonged incubation with 200 mM sucrose; the percentage of dead cells after 2hr and 4hr incubation with 200 mM sucrose was 1.5 and 1.9 fold higher (respectively) than in control samples (Fig 3). These changes were statistically increased from control, but there was no significance between the two time points of 2 and 4 hr. The DNA fragmentation fluorescent microscopy assay showed that after 4hr hypertonic exposure (200mM sucrose) 6±1% (n = 5) of cells had a bright green nucleus with condensed chromatin, consistent with the development of DNA fragmentation.

## Discussion

In this report we investigated whether hypertonic challenge triggers cell death and/or regulatory volume increase (RVI) in the human skeletal muscle cell line, TE671RD. We found that a significant proportion of cells underwent RVI and there was minimal resultant cell death even with very high osmotic challenge. Initial shrinkage very closely fitted that expected from the “ideal osmotic shrinkage” described in native skeletal muscle fibres by Blink *et al* [38] and Ferenczi *et al* [39]. Blink *et al* [38] saw little evidence of RVI and Ferenczi *et al* [39] reported only a few percent RVI. In contrast, Lindinger *et al* saw significant and rapid RVI in mouse soleus (slow twitch) skeletal muscle [40]. We find that the TE671RD population divides in to subpopulations with and without significant RVI capacity in a similar way to that we reported previously for leukaemia U937 cells [2].

Apoptosis is a stereotyped form of programmed cell death which includes a number of morphological and biochemical changes including DNA fragmentation [41]. The degree of increased osmolality in older people is relatively small (3%) [13], compared with the challenge we applied here of up to 200mOsm/Kg H_2_0, but in our assays only a small proportion of cells showed evidence of DNA fragmentation. We would therefore tend to *exclude* increased osmolality as a potential trigger for apoptosis and sarcopenia in ageing people. It should be noted, however, that we applied a high osmolality solution for only a few hours, whilst naturally, people would have their small elevation in plasma osmolality for many years. The osmolalities we used here were quite extreme; (DMEM plus added sucrose) varied between 300 and 500mOsm/Kg H_2_O. It was shown in rabbit studies that 400mOsm/Kg H_2_O and above results in severe clinical symptoms, and most animals died by 450mOsm/Kg H_2_O [42].

One particular point of note in the present study is the apparently slow rate of shrinkage, with minimum volume being measured at a time of 2hrs following hypertonic challenge. There are several points surrounding this. The first is that since our protocol was to measure at 0.5 hrs and then again at 2 hrs, it is entirely possible that the minimum volume was actually reached well before 2 hrs. The reason for this is that our experimental design was not intended to demonstrate the kinetic properties and follow progression of individual cells (as we have done in some other studies), but to follow populations of cells. Some cells trigger RVI earlier, and some later (some not at all), but we felt that by 2 hrs all cells that were going to undergo RVI would have begun to do so. We caution against kinetic interpretation of our data, however, since the study simply was not designed for that. A more interesting hypothesis is that these cells have very few aquaporin channels and very low water permeability. We will investigate this in the future. From a biological/biochemical perspective it is interesting to note that the rate of attaining a new cellular volume following change of solution osmolality is dependent upon cell water permeability. This can be at least 10 fold different between different types of cells and is greatly affected by the composition of the extracellular solution [43]. In cases where similarly slow rates of cellular shrinkage have been observed [2, 43, 44], the extracellular osmolality was also increased by sucrose (similar to our own study) which seems to decrease cell permeability [43]. One might speculate this could involve block of aquaporin channels by sucrose, but we are not aware if this has been investigated directly. Yurinskaya *et al* [2] show comparative shrinkage to be much faster in the presence of elevated NaCl compared to sucrose and the rapid skeletal muscle shrinkage observed by [40] used addition of NaCl. Whilst the aquaporin composition of skeletal muscle varies with age [17], the predominant isoform is AQP4 [45] and further pharmacological analysis of this and other aquaporins would be useful. Finally, it is possible that following passive shrinkage, a degree of active volume shrinkage then takes place; the mechanism of which would be beyond the scope of the current manuscript. This could be some sort of “dying cell volume decrease” (DVD) as seem in many [4, 5], but not all cell types [4, 46]. Since we did not see a great deal of cell death itself, even at 4 hrs, this seems unlikely. We found that a rather high proportion of cells lacked RVI, but few cells appeared to initiate apoptosis and so our data does not support a notion that hypertonic shrinkage and lack of RVI directly correlates to induction of apoptosis. This does not directly shed light on the debate as to whether shrinkage is a necessary step for apoptosis in this cell line, however [4, 5], since there was so little evidence of apoptosis itself.

The present study used a human skeletal muscle derived cell line, TE671RD. Previously, TE671RD cells have been used mainly for investigation of the human nicotinic receptor physiology [33], but we will continue to investigate its volume regulatory properties in parallel with other *in vitro* skeletal muscle systems. Myoblasts formed from freshly isolated rodent tissue would be an alternative skeletal muscle cell volume model and it would be interesting to compare the volume regulatory properties of human TE671RD cells to such cell lines as the rat L6 or mouse C2C12. The attraction to us of TE671RD cells is that they constitute a human skeletal muscle derived cell line and thus express human muscle isoforms of important muscle receptors such as the nicotinic acetylcholine receptor [25]. There is unlikely to be a single perfect *in vitro* model of skeletal muscle cell volume control. For example, in the introduction to this manuscript we discuss known similarities and differences between native skeletal muscle and TE671RD cells, but it is also likely that ion channel expression is dissimilar between different skeletal muscle fibre types within *the same* species. In terms of volume regulation, TE671RD cells form round cells and allow for the measurement of cellular volume from 2D surface area in a way difficult to reproduce with freshly dissociated skeletal muscle, but possible with the rodent myoblasts. The clear limitation is the differences we have observed (above) between TE671RD cells and previously reported native skeletal muscle. In future studies we will continue to investigate the cellular machinery of skeletal muscle cellular volume control, both in terms of its regulatory volume increases and decreases. Furthermore, we will explore apoptosis in this cell line and investigate responses to inflammatory cytokines known to be important for skeletal muscle senescence. This work may prove useful, not just in the study of ageing, but also in the disease most closely thought to involve skeletal muscle cellular volume control; compartment syndrome.

In conclusion, we found that the human skeletal muscle cell line, TE671RD exhibits greater active cell volume regulation than reported for some native skeletal muscle and the cells are very resistant to hypertonicity triggered cell death. If these effects were reproduced in native human skeletal muscle it would strongly suggest that the relatively modest plasma hypertonicity measured in older people is not likely to contribute to cell death or sarcopenia.
